# Supplementary material for: “If you miss that first step in the chain of survival, there is no second step”–Emergency ambulance call-takers’ experiences in managing out-of-hospital cardiac arrest calls
Source: PLoS One. 2023 Mar 13;18(3):e0279521. doi: 10.1371/journal.pone.0279521 (PMC10010558; doi:10.1371/journal.pone.0279521)
Supplement: S1 File — (DOCX) [file pone.0279521.s001.docx]

# **Supplementary material**

## **Interview format**

Since the interview was designed to be open-ended, these questions were used as a guide to prompt the participant to speak, therefore superfluous questions could were omitted in some interviews.

So, (insert name), this interview is about your experiences with OHCA calls. It’s an open interview so I want to encourage you to talk at length about the topics, so please take your time. There are no right or wrong answers. I really want to hear your opinions based on your own experiences.

1. To start off, can you tell me how you became a call-taker at SJ-WA? (warm-up question to build rapport)
2. How often do you take calls where the patient is in cardiac arrest? (priming question)
3. What comes into your mind when you determine that an OHCA has happened or is happening during the call? (general question)
4. Can you talk about how the call process generally goes for OHCA calls? (general question)
5. Can you tell me about your experiences using MPDS for OHCA calls? I want to hear anything – things that help, things that don’t work so well, how you view MPDS (specific question)
6. How do you find working with ProQA during OHCA calls – how does it help or hinder you during the OHCA call? (specific question)
7. How does MPDS work for you when it comes to getting the defibrillator? (specific question)
8. How does MPDS work for you when it comes to getting the bystander to do CPR?
9. Are there any challenging parts for you in OHCA calls? How do you experience them and handle them? (specific question)
10. For the final question, I want you to imagine you are the manager of this State Operations Centre and you get to direct call-takers on how they should handle OHCA calls. You can give them advice on how to use MPDS and ProQA, you can make new rules about call procedures, you can give them tips on how to best manage an OHCA call. What would you say? (empowering question to close the interview)
