# Supplementary material for: “If you miss that first step in the chain of survival, there is no second step”–Emergency ambulance call-takers’ experiences in managing out-of-hospital cardiac arrest calls
Source: PLoS One. 2023 Mar 13;18(3):e0279521. doi: 10.1371/journal.pone.0279521 (PMC10010558; doi:10.1371/journal.pone.0279521)
Supplement: S2 File — (PDF) [file pone.0279521.s002.pdf]

Research project

Curtin University

PREHOSPITAL, RESUSCITATION AND EMERGENCY CARE RESEARCH UNIT

To St John WA comms officers

Please consider taking part in this research project by PRECRU at Curtin University in 2021

Exploring the experiences of ambulance call-centre staff in managing out of hospital cardiac arrest (OHCA) "000" emergency calls

As you know, cardiac arrest is one of the most critical emergencies. We want to ask you about your experiences of handling cardiac arrest calls. We are keen to find out things like:

- Whether the MPDS protocol for OHCA works well for you
- Whether there are things that could be improved when it comes to handling OHCA calls
- The things that EMD experience with OHCA calls that the rest of us are not aware of.

We'd like to do a one hour interview with you (during your shift, and approved by SJ-WA). Your input will be confidential – that is, we won't connect your name to what you say in any public forum. We don't intend to ask you any personal or sensitive questions – we just want to talk about call-taking processes for OHCA.

For more information, please contact Dr Niru Perera (pictured) at niru.perera@curtin.edu.au or on XXX

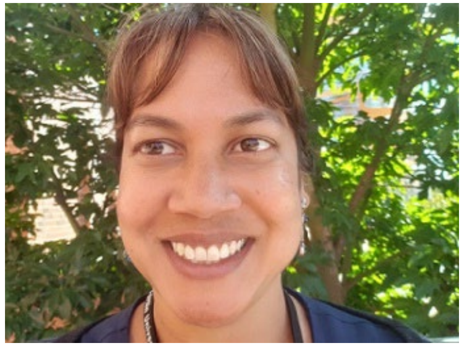

More details will follow next month…

To St John WA Comms Officers

Please participate in our research interviews this June

PRECRU research project

Exploring the experiences of ambulance call-centre staff in managing out of hospital cardiac arrest (OHCA) "000" emergency calls

For more information, please contact Dr Niru Perera (pictured), Research Fellow at PRECRU at niru.perera@curtin.edu.au or on XXX

Curtin University Human Research Ethics Committee (HREC) has approved this study (HRE2021-0008)

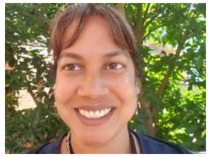

Curtin University

PREHOSPITAL, RESUSCITATION AND EMERGENCY CARE RESEARCH UNIT

Questions and Answers

1. Is this part of my job?

No. This is voluntary. You will be asked to do an interview during work time and this has been approved by St John WA. But being involved in the project is not compulsory.

2. What do I have to do?

Niru, the lead researcher, will have a one-on-one interview with you in a private office at St John WA in Belmont. The interview will last about an hour. That's the main part of your involvement. After that, Niru will email you for your input on the results of the interviews. Your involvement in the project is expected to be complete by December 2021.

3. Will anyone at St John find out what I said?

No, your identity will be hidden. No-one (except Niru) will know what you said in the interviews. The PRECRU study investigators (Judith Finn, Stephen Ball & Tanya Birnie) will have access to the interview recordings but these will not have your name attached to them. No staff at St John WA will have access to the interview recordings or any interview data that identifies you.

4. Are there going to be any personal questions?

No, the questions are going to be professional – mainly about how you manage OHCA calls, how you find using MPDS and ProQA, whether any processes could be improved. I won't ask you to talk about your personal life or feelings and I won't ask you about any of your colleagues or management at St John WA. However, if you do need any support during or after the interview, you can see the SJ-WA support crew.

5. Will I benefit from the research?

There will not be any direct benefit for you. However there may be indirect benefits because what you say in the interviews can help us to make recommendations for improving the OHCA call call-taking process.

Curtin University

PREHOSPITAL, RESUSCITATION AND EMERGENCY CARE RESEARCH UNIT

PRECRU research project: Exploring the experiences of ambulance call-centre staff in managing out of hospital cardiac arrest (OHCA) “000” emergency calls

We are still calling for volunteers

- We are looking for about 20 comms officers to volunteer in our research project.
- We will ask you to attend a one-hour interview during your shift. We want to ask you questions about cardiac arrest calls – what’s been your experience of them? Does the MPDS process work well?
- Your participation is completely voluntary and you are under no obligation to take part.

What to do next

Dr Niru Perera is a research fellow in linguistic analysis at PRECRU. She will be doing the interviews and is keen to understand cardiac arrest call communication from comms officers’ point of view. She has more information about the project and can answer any questions.

Please contact her: niru.perera@curtin.edu.au or on XXX for an obligation-free chat.

Then you can decide whether you want to take part.

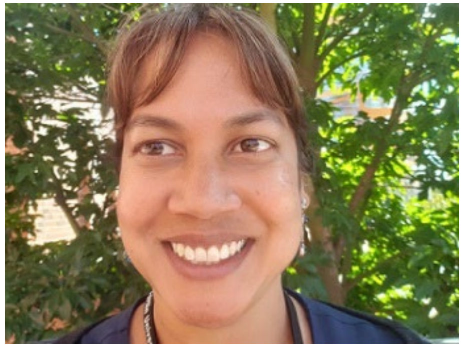

Curtin University Human Research Ethics Committee (HREC) has approved this study (HREC number HRE2021-0008)

To St John WA Comms Officers

Please participate in our research interviews this June

PRECRU research project

Exploring the experiences of ambulance call-centre staff in managing out of hospital cardiac arrest (OHCA) “000” emergency calls

For more information, please contact Dr Niru Perera (pictured), Research Fellow at PRECRU at niru.perera@curtin.edu.au or on XXX

Curtin University Human Research Ethics Committee (HREC) has approved this study (HRE2021-0008)

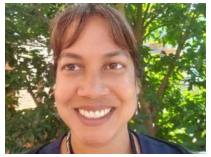

Curtin University
PREHOSPITAL, RESUSCITATION AND EMERGENCY CARE RESEARCH UNIT

Questions and Answers

1. Is this part of my job?

No. This is voluntary. You will be asked to do an interview during work time and this has been approved by St John WA. But being involved in the project is not compulsory.

2. What do I have to do?

Niru, the lead researcher, will have a one-on-one interview with you in a private office at St John WA in Belmont. The interview will last about an hour. That’s the main part of your involvement. After that, Niru will email you for your input on the results of the interviews. Your involvement in the project is expected to be complete by December 2021.

3. Will anyone at St John find out what I said?

No, your identity will be hidden. No-one (except Niru) will know what you said in the interviews. The PRECRU study investigators (Judith Finn, Stephen Ball & Tanya Birnie) will have access to the interview recordings but these will not have your name attached to them. No staff at St John WA will have access to the interview recordings or any interview data that identifies you.

4. Are there going to be any personal questions?

No, the questions are going to be professional – mainly about how you manage OHCA calls, how you find using MPDS and ProQA, whether any processes could be improved. I won’t ask you to talk about your personal life or feelings and I won’t ask you about any of your colleagues or management at St John WA. However, if you do need any support during or after the interview, you can see the SJ-WA support crew.

5. Will I benefit from the research?

There will not be any direct benefit for you. However there may be indirect benefits because what you say in the interviews can help us to make recommendations for improving the OHCA call call-taking process.
