# Supplementary material for: “If you miss that first step in the chain of survival, there is no second step”–Emergency ambulance call-takers’ experiences in managing out-of-hospital cardiac arrest calls
Source: PLoS One. 2023 Mar 13;18(3):e0279521. doi: 10.1371/journal.pone.0279521 (PMC10010558; doi:10.1371/journal.pone.0279521)
Supplement: S3 File — (DOCX) [file pone.0279521.s003.docx]

# **Supplementary material**

## **Findings**

In this section, further interview excerpts are provided around the sub-theme of cardiopulmonary resuscitation, specifically, how call-takers managed the quality of cardiopulmonary resuscitation (CPR).

### **The dispatch process/Cardiopulmonary resuscitation/CPR quality management**

When it came to ensuring that compressions were consistent and effective, call-takers took a few different approaches. One approach was to prioritise commencing CPR and then focussing on the quality aspect once the bystander got into a rhythm.

Whereas it's all about, let's just start the CPR straightaway. And maybe, then we fine tune what they're actually doing. Because I guess some, some bad CPR is better than no CPR. (Buddy)

Another aspect of quality management of CPR was the encouragement that call-takers gave to motivate bystanders to continue, as time moved on and tiredness set in. One key strategy was to direct bystanders to take a break and swap with another, if this option was available.

You just gonna support and encourage and really try and push them through those compressions. But if they've got other people there with them, you know, you can get them swapping over, check in regularly to make sure they're doing the compressions at the right speed and in the right way. (Deb)

As part of CPR quality management, the ProQA system helped call-takers to track the number of compressions. However, call-takers displayed different approaches to counting compressions aloud for the caller. While it was seen as helpful, call-takers acknowledged that, for long sessions of CPR, it could become exhausting for the call-taker and perhaps not the most effective use of their time.

Some of the call takers will do the counting with them… something I've always done, I [only] count with them at the start, like ‘follow my voice, and we'll do the 1-2-3-4’. And then I'll stop maybe after the fourth or fifth cycle. And I'll still tell them to count because that way I can hear, that's the only reference I've got to make sure that they're doing the timing correctly. And then that way, while they're doing the counting, I can take a step back, I know they're doing well. And then I can maybe tidy up the call card, send some information to the crew. I can speak to them while they're doing the counting. (Buddy)

Some call-takers mentioned using a metronome to help with the counting of compressions. However Cody suggested that this could be another area for improvement by embedding the sound of a metronome into the call system, thus it could not be heard by other call-takers in the room.

I think for us to have that built into our system … so they could hear that beeping … rather than us going ‘1234’. We could still say ‘okay, you're doing really, really well’. But in the background, just that little beeping, just get that rhythm going. And then the rest of the room don't hear it. It's only the caller can hear on their phone, it's beeping on their phone. It's not interrupting anything going on. (Cody)
